# Fetal bovine serum, an important factor affecting the reproducibility of cell experiments

**DOI:** 10.1038/s41598-023-29060-7

**Published:** 2023-02-02

**Authors:** Shuai Liu, Wei Yang, Yunlei Li, Changqing Sun

**Affiliations:** 1 Department of Laboratory, Shenzhen Samii International Medical Center (Shenzhen Fourth People’s Hospital), Shenzhen, 518118 People’s Republic of China; 2 Institute of Biomedical Engineering, Shenzhen People’s Hospital (The Second Clinical Medical College, Jinan University; The First Affiliated Hospital, Southern University of Science and Technology), Shenzhen, 518020 Guangdong People’s Republic of China; 3 Department of Clinical Laboratory, Shenzhen Traditional Chinese Medicine Hospital, The Fourth Clinical Medical College of Guangzhou University of Chinese Medicine, Shenzhen, 518033 People’s Republic of China

**Keywords:** Cytokines, Cell biology

## Abstract

Fetal bovine serum (FBS) is a natural medium used in cell cultures containing the large amount of nutrients necessary for cell growth and is often used for in vitro cultures of animal cells. Although FBS plays a vital role in cell cultures, there are small molecules contained within FBS that remain unidentified, and their effects on cultured cells is poorly understood. Here, we report that different brands of FBS have varying influences on the background expression of IL-8, not TNFα and IL1β in epithelial cells. The endogenous small molecules in FBS and ERK pathways may contribute to these effects. In addition, FBS form the IL-8 stimulation and IL-8 non-responsive groups have different metabolome profiles. Overall, our study suggests that metabolites in FBS should be included in the quantitative considerations when conducting cell experiments, especially immune-related experiments, to improve the repeatability of experimental results in scientific papers; IL-8 could thus be an important factor in selecting FBS.

## Introduction

As science and technology continue to develop, massive amounts of data are generated by scientific activities around the world, further facilitating this development. However, Baker reported that nearly 80% of biologists cannot repeat the results of others’ experiments, and 60% cannot repeat their own experimental results^1^. Although various field-specific methods have been adopted to improve the repeatability of scientific research, repeatability remains restricted by a variety of objective factors.

Animal cell cultures facilitate in vitro experimental methods, simulating the intracorporeal environment, as well as maintaining its own main structure and function in cellular survival, growth, and proliferation. The use of animal cell cultures is an important and commonly used technology in cell biology research and can be used as a platform for oncology and basic research in immunology and other disciplines. The in vitro culture of animal cells is inseparable from the use of serum, particularly fetal bovine serum (FBS). However, there is no standard method to evaluate the potential adverse effects on an experiment due to unknown factors in the complex composition of FBS^2–6^.

Although some studies have attempted to use known ingredients to replace FBS^7–9^, the efficacy of FBS in cell cultures remains incomparable. The ingredients and origins of FBS from different batches and brands are unknown to their users, and no recognized standard for cell cultures exists. This affects the uniformity of cell culture effects among different laboratories and undoubtedly affects the reproducibility of experimental results. Many journals have ignored the potential impact of serum variations on cell cultures and have not required researchers to indicate the brand and batch of serum used in their work. In the present study, we selected eight brands of FBS originating from three producing regions with high frequency of usage and good brand acceptance to culture epithelial cells and analyze the influence of serum factors on the basic expression of inflammatory factors. In doing so, we sought to preliminarily evaluate the influence of different brands of serum.

## Results

### Effects on IL-8 secretion in epithelial cells differ among FBS brands

**Figure 1:**
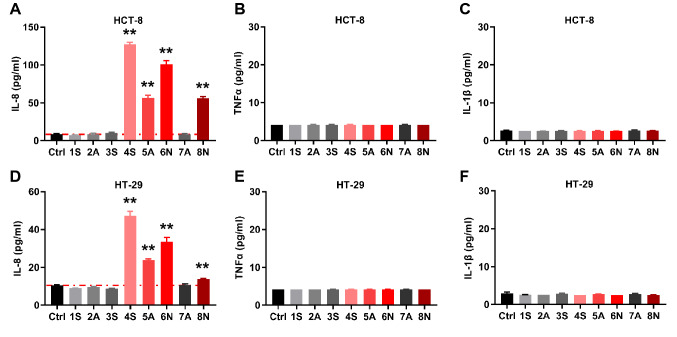
Different brands of FBS affect IL-8 secretion in HCT-8 and HT-29 cells. Cultured HCT-8 and HT-29 cells were serum-starved overnight and treated with 10% FBS for 5 h; the medium was then collected, and IL-8, TNFα, and IL-1β levels were detected by ELISA. (**A**) IL-8 secretion from HCT-8. (**B**) TNFα secretion from HCT-8. (**C.** IL-1β secretion from HCT-8. (**D.** IL-8 secretion from HT-29. (**E**) TNFα secretion from HT-29. (**F**) IL-1β secretion from HT-29. Data is presented by mean ± SEM of three independent experiments. **P < 0.01, significantly different from the control (Ctrl) group.

To investigate the influence of different brands of FBS on cultured cells, eight high-frequency serum brands originating from South America, Australia, and New Zealand (see Table 1 for details) were selected to culture epithelial cells and analyze the expression levels of inflammatory factors. The results showed that the 4S, 5A, 6N, and 8N FBS significantly induced the secretion of IL-8 in HCT-8 cells (Fig. 1A) but did not cause the secretion of TNFα and IL-1β (Fig. 1B,C). However, 1S, 2A, 3S, and 7A FBS had no effect on the secretion of IL-8, TNFα, and IL-1β. This was consistent with the results for the HT-29 cells (Fig. 1D,E,F). These results also indicated that there were certain factors in the 4S, 5A, 6N, and 8N FBS that induced epithelial cells to promote the expression of IL-8.

**Table 1:** Fetal Bovine Serum (FBS) from different brands was used in this study. Cat., Catalogue number; Lot., Lot Number.

	Brand	Cat	Lot	Origin
1S	Excell Bio	FSP500	12J068	South America
2A	Excell Bio	FND500	11I249	Australia
3S	Biological Industries	04-001-1ACS	2001003	South America
4S	Gibco	10270-106	2232237	South America
5A	Gibco	10099-141	2168090RP	Australia
6N	Corning	35-081-CV	35081002	New Zealand
7A	Hyclone	SV30308.02	SF0007003	Australia
8N	Bovogen	SFBS	1907B	New Zealand

### Small molecules in FBS promote the expression of IL-8 by activating the pERK pathway

**Figure 2:**
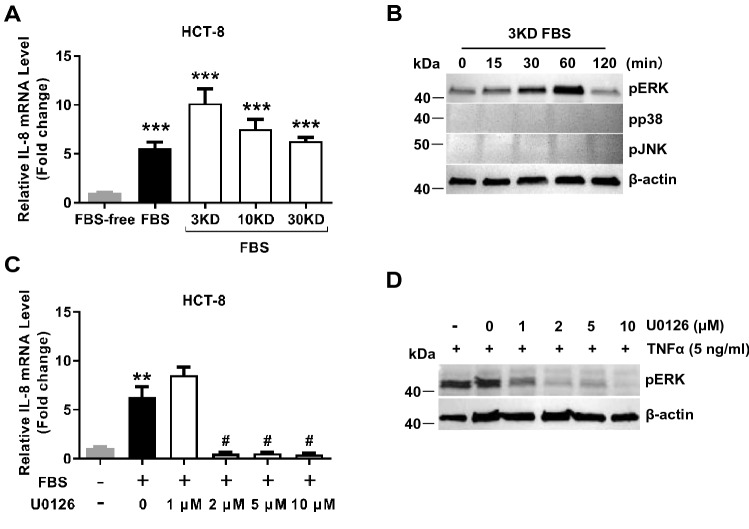
Small molecules in FBS promoting IL-8 expression via ERK activation in HCT-8 cells. (**A**) The whole FBS (Biological Industries) was separated into a protein fraction (< 3KD, < 10KD, or < 30KD) by using a series of centrifugal spin filters, then to treat HCT-8 for 5 h, respectively. The IL-8 level was detected by QPCR. (**B**) HCT8 cells were serum-starved overnight and treated with 3KD FBS (Biological Industries) for the indicated time, then intracellular pERK, pp38, and pJNK were analyzed by western blot. β-actin was an internal control. Full-length gels are presented in Supplementary Fig. 1. (**C**) The pERK inhibitor U0126 can abolish the expression of IL-8 induced by FBS (Biological Industries). (**D**) The effect of the U0126 (pERK inhibitor) treatment was verified by western blot. Full-length gels are presented in Supplementary Fig. 1. The pERK was activated by 5 ng/ml TNFα as a positive control. Data is presented by mean ± SEM of three independent experiments. **P < 0.01, ***P < 0.001 indicate significantly different from the control group; # indicates significance compared to FBS-only treatment.

To verify that the substance promoting the secretion of IL-8 in epithelial cells, ultrafiltration was used to separate FBS components based on molecular weights, including < 3KD, < 10KD, and < 30KD components. Results showed that the < 3KD, < 10KD, and < 30KD components significantly promoted the level of IL-8 mRNA in the HCT-8 cells (Fig. 2A). The < 3KD components had the most significant effect, suggesting that the small molecules contained in the serum may play a role in promotion. Upon further analysis, we found that the < 3KD components of the FBS activated pERK in the HCT-8 cells without affecting the levels of pp38 and pJNK (Fig. 2B). Consistent with this, U0126, a pERK specific inhibitor, was shown to abolish the induction of IL-8 mRNA expression by the < 3KD FBS components (Fig. 2C), showing that the < 3KD FBS components induced IL-8 mRNA expression in the HCT-8 cells by activating the pERK pathway (Fig. 2D).

### Metabolome profiles differ among brands of FBS

**Figure 3:**
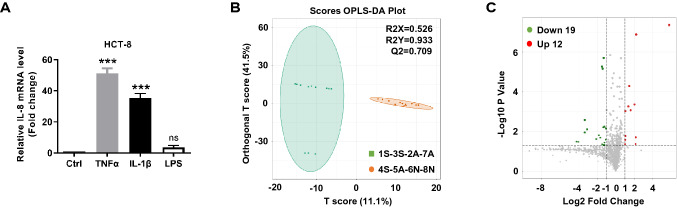
Differences in endogenous metabolites in serum of different brands. (**A**) HCT-8 cells were serum-starved overnight and treated with TNFα (2 ng/ml), IL-1β (2 ng/ml), and LPS (5 ng/ml) for 2 h, then IL-8 level was analyzed by QPCR. (**B**) Orthogonal least squares–discriminant analysis (OPLS-DA) score plot of the two groups of FBS was performed by using R (package MetaboAnalystR, version 1.0.1) software. **C.** Volcano plot indicated up-regulated and down-regulated metabolites in two groups of FBS. Data is presented by mean ± SEM of three independent experiments. ***P < 0.001, significantly different from the control (Ctrl) group.

**Figure 4:**
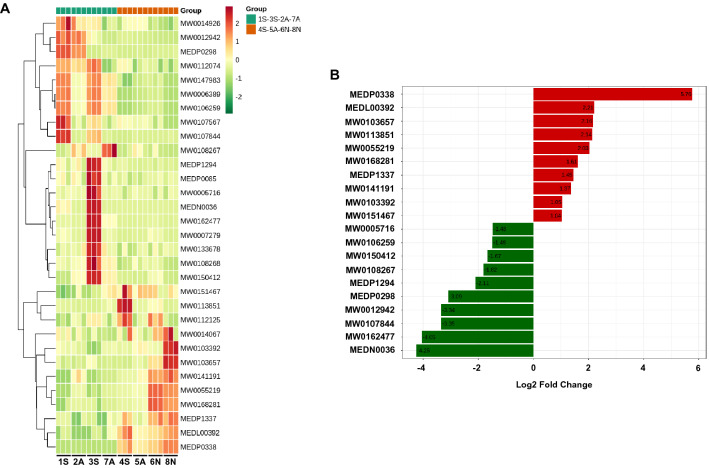
Metabolic differences between the two cohorts. (**A**) Heatmap showed hierarchical clustering of different metabolites in two groups of FBS was performed by using R (package pheatmap, version 1.0.12) software. Up-regulated and down-regulated metabolites are colored in red and green, respectively, indicating the results of the cluster analysis. (**B**) Bar graph presenting the top 10 up-regulated and down-regulated metabolites.

**Figure 5:**
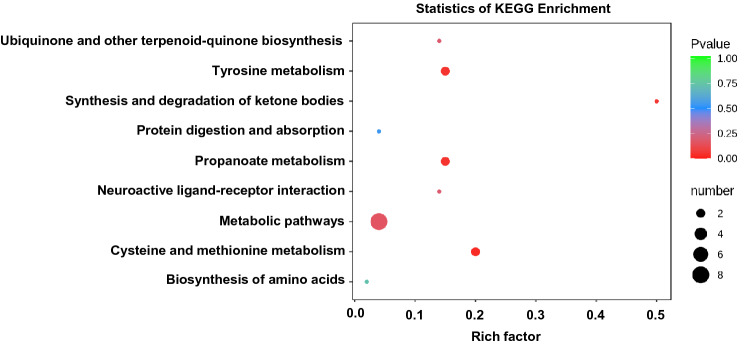
KEGG pathway analysis. Metabolites enriched KEGG pathway^10^ scatterplot showing statistics of pathway enrichment in two groups of FBS.

Because IL-8 can be induced by IL-1β and TNFα instead of LPS in HCT-8 cells (Fig. 3A), and 4S, 5A, 6N, and 8N FBS did not induce the secretion of IL-1β and TNFα, we speculated that it was the endogenous substance that induced the secretion of IL-8 in FBS, not the exogenous endotoxin substance. To this end, we divided eight brands of FBS into two groups—the IL-8 stimulation group (4S, 5A, 6N, and 8N) and the IL-8 non-responsive group (1S, 2A, 3S, and 7A)—and used non-targeted metabolomics to analyze the endogenous metabolites in all brands of FBS. The principal component analysis showed that 4S, 5A, 6N, and 8N FBS were quite different from the 1S, 2A, 3S, and 7A FBS, but their intra-group variability was low (Fig. 3B). Compared to the IL-8 non-responsive group, 12 metabolites in the IL-8 stimulation group were up-regulated, and 19 metabolites were down-regulated (Fig. 3C). Notably, the up-regulated metabolites were MEDP0338, MEDL00392, MW0103657, MW0113851, MW0055219, MW0168281, MEDP1337, MW0141191, MW0103392, MW0151467, MW0014067, and MW0112125 (Fig. 4A), of which 1-Palmitoyl-sn-glycero-3-phosphocholine (MEDP0338) was almost non-existent in the serum of the IL-8 non-responsive group; however, its abundance was higher in the serum of the IL-8 stimulation group, which increased by 54.28 times (Fig. 4B; see Supplementary material for specific information on differential metabolites).

### Differential metabolites originated in amino acid metabolism

Further analysis of the causes of differences in metabolites in FBS was done. KEGG analysis results showed that differential metabolites originated in the metabolism of amino acid, tyrosine, cysteine, and methionine, among others (Fig. 5).

## Materials and methods

### Fetal bovine serum

Eight brands of fetal bovine serum (FBS) were obtained from ExCell Bio (origin: South America; Cat.: FSP500; Lot.: 12J068; Shanghai, China), ExCell Bio (origin: Australia; Cat.: FND500; Lot.: 11I249; Shanghai, China), Biological Industries (origin: South America; Cat.: 04-001-1ACS; Lot.:2001003; Kibbutz Beit-Haemek, Israel), Gibco (origin: South America; Cat.: 10270-106; Lot.: 2232237; Paisley, UK), Gibco (origin: Australia; Cat.: 10099-141; Lot.: 2168090RP; Paisley, UK), Corning (origin: New Zealand; Cat.: 35-081-CV; Lot.: 35081002; NY, USA), Hyclone (origin: Australia; Cat.: SV30308.02; Lot.: SF0007003; Logan, USA), Bovogen (origin: New Zealand; Cat.: SFBS; Lot.: 1907B; Melbourne, Australia).

### Cell culture

The HCT-8 and HT-29 cells were obtained from ATCC by vendors and were validated further using short tandem repeat (STR) genotyping. Cells were grown in Dulbecco's Modified Eagle Medium (DMEM) with 10% FBS and 1 × penicillin/streptomycin. All cells were grown at 37 °C in a 5% CO_2_ incubator. The cells were regularly tested for potential mycoplasma contamination using the Myco-Blue Mycoplasma Detector (Vazyme, D101, Nanjing, China).

### Real-time quantitative polymerase chain reaction

Total RNA was extracted using NucleZOL (Macherey Nagel, Düren, Germany) according to the manufacturer’s instructions. A total RNA of 1 μg was used for reverse transcription performed via HiScript® III RT SuperMix with a gDNA wiper (R323, Vazyme). Real-time PCR was performed using the ChamQ Universal SYBR qPCR Master Mix (Q711, Vazyme). β-actin was used in endogenous control transcripts for normalization of the target transcripts. The relative gene expression was analyzed using the 2-∆∆Ct method. The following primers were used: Human *IL-8* (forward: CTTGGCAGCCTTCCTGATTT; reverse: TTCCTTGGGGTCCAGACAGA), internal control *β-actin* (forward: ACTGGAACGGTGAAGGTGAC; reverse: AGAGAAGTGGGGTGGCTTTT).

### Enzyme-linked immunosorbent assay

Approximately 1 × 10^5^ HCT-8 or HT-29 cells were plated in a 24-well plate and were then serum-starved overnight and treated with 10% FBS for 5 h. The culture supernatant was collected, and IL-1β, IL-8, and TNFα were detected using IL-8 (88-8086-86, Thermo Scientific), IL-1β, (88-7261-88, Thermo Scientific), and TNFα (88-7346-88, Thermo Scientific) kits, respectively, according to the manufacturer’s instructions.

### Sample preparation and extraction

Samples were thawed on ice, and 3 volumes of ice-cold methanol were added to 1 volume of serum; the mixture was whirled for 2 min and incubated at −20 °C for 0.5 h. The mixture was then whirled for 2 min and centrifuged at 12,000 rpm at 4 °C for 10 min. The supernatant was collected and incubated at −20 °C for 0.5 h. Finally, the mixture was centrifuged at 12,000 rpm at 4 °C for 15 min, and the supernatant was collected again for LC–MS/MS analysis.

### HPLC conditions (T3)

All samples were acquired using the Liquid Chromatography Mass Spectrometry (LC–MS) system and following machine orders. The analytical conditions were as follows: UPLC column, Waters ACQUITY UPLC HSS T3 C18 (1.8 µm, 2.1 mm * 100 mm); column temperature, 40 ℃; flow rate, 0.4 mL/min; injection volume, 2 μL; solvent system, water (0.1% formic acid): acetonitrile (0.1% formic acid); and gradient program, 95:5 V/V at 0 min, 10:90 V/V at 11.0 min, 10:90 V/V at 12.0 min, 95:5 V/V at 12.1 min, and 95:5 V/V at 14.0 min.

### Metabonomic analysis

The original data file obtained by LC–MS analysis was first converted into the mzML format using ProteoWizard software (Version 3.0.19095-938eda31a, http://proteowizard.sourceforge.net). Peak extraction, alignment, and retention time correction were performed using the XCMS program. The SVR method was used to correct the peak area. Filter the peaks with deletion rate > 50% in each group of samples. Next, metabolic identification information was obtained by searching the laboratory’s self-built database and integrating the public database and metDNA. Finally, statistical analysis was carried out using the R program. Statistical analysis included univariate analysis and multivariate analysis. Univariate statistical analysis included the Student’s *t* test and variance multiple analysis. Multivariate statistical analysis included principal component analysis (PCA), partial least squares–discriminant analysis (PLS-DA), and orthogonal partial least squares–discriminant analysis (OPLS-DA).

### Immunoblotting

The cells were treated as described above and were collected by centrifugation with 500*g* for 5 min, then washed twice in cold PBS and lysed with RIPA Lysis Buffer (Beyotime, Shanghai, China). The lysates were quantified using a BCA protein quantification kit (Yeasen). The proteins were separated by SDS-PAGE, immunoblotted, and visualized using the Tanon 5200 Chemiluminescent Imaging System (Tanon Science & Technology), then the images were cropped by Corel DRAW X3 SP2 software (https://www.coreldraw.com/en/pages/coreldraw-x3/).

### Separation of FBS components

FBS components 3 KD, 10 KD, and 30 KD were isolated using a series of centrifugal spin filters. Molecular-weight cutoff (MWCO) of 3 KD (#88525), 10 KD (#88527), and 30 KD (#88529) Pierce™ Protein Concentrators PES was obtained from Thermo Fisher Scientific (Cleveland, OH, USA).

### Statistical analysis

All data was presented as mean ± SEM. The statistical software GraphPad Prism 7.0 was used for data analysis. Comparison between only two groups was conducted by two-tailed unpaired Student’s t test; comparisons among three or even more groups were conducted by ANOVA with post-hoc test (multiple comparisons, one-way ANOVA for one categorical independent variable, while two-way ANOVA for two categorical independent variables). Differences were considered statistically significant as described.

## Discussion

For many years, the repeatability of experimental results has been an important aspect affecting the development of scientific research^1^. Much research data was generated with cancer cell lines as the research object, which has been the basis for most subsequent research. However, cell line confusion caused by misidentification, cross-contamination, poor annotation^11,12^, and mycoplasma infection^13–16^, among other causes, led to the irreproducibility of experimental results among different laboratories. This means that considerable funding for scientific research has been wasted. To this end, researchers have used biochips to detect changes in the expression of hundreds of genes in cells, including gene encoding receptors, ion channels, growth factors, and oncogenes, caused by mycoplasma infection^14^. Mycoplasma contains highly immunogenic lipoproteins, which are anchored on the outer surface of the cell membrane to be recognized by the receptors of immune cells, such as TLR2, to activate the NF-κB pathway and further lead to abnormal cellular activation and final deviation of experimental results^17^. Recently, Liu et al. found that the degree to which genotype variability induced protein type and phenotypic variation in the same cell line cultured in different laboratories is an important factor contributing to the irreproducibility of experimental results^18^. Therefore, continuing to investigate factors leading to unrepeatable experimental results is crucial in the development of scientific research.

FBS is a mixture formed by removing fibrin from fetal bovine plasma, which contains various plasma proteins, peptides, fats, growth factors, hormones, inorganic substances and some unclear small molecules, including different kinds and concentrations of metabolites that may have great effects on the biological behavioral and stress response of cultured cells^19^. FBS is a natural medium used in cell cultures with the large amount of nutrients necessary for the in vitro culture of animal cells Moreover, FBS content and type often vary with the sex, age, physiological conditions, and nutritional conditions of the blood supply animal. The climate of the place of FBS production may also affect FBS makeup^20,21^. Every laboratory has unique experiences selecting ideal FBS, often considering the price of the serum and the stability of the cultured cells. It is often overlooked that FBS itself may have differences in the background expression of certain genes in cells, and there are no authoritative studies for analysis and comparison.

Although there are some other supplements for cell culture, such as human platelet lysates (hPL)^22^, considering the high price of hPL, FBS is still the common supplement used for cell culture in most laboratory throughout the world. For repeatability of experimental data, it is important to point out, especially for non-immune-related researchers, that supplement with high inflammatory factors will significantly alter the stress response of static culture cells. The choice of FBS must be carefully considered, and it is meaningful to be stated in current publications. In addition, according to our results, the endogenous small molecule metabolites in FBS have obvious impacts on cellular inflammatory response, which is independent of species but may be influenced by the diet, the growth environment and individual variations of animals. In terms of endogenous metabolites, even hPL may also have the same problem^22^. Therefore, we examine the variations in the FBS of several brands from three origins in this article.

In this study, we found that different brands of FBS had varying influences on the background expression of IL-8, an inflammatory factor that has been extensively studied, but not on the expression of IL-1β and TNFα in epithelial cells. Metabolome analysis indicated that the IL-8 stimulation group of FBS and the IL-8 non-responsive group of FBS had different metabolite profiles, with 12 up-regulation and 19 down-regulation, respectively. The differences in FBS metabolites between the two groups were mainly caused by amino acid metabolism, of which 1-Palmitoyl-sn-glycero-3-phosphocholine (MEDP0338) was the most remarkable. In addition, the small molecular components in FBS that were less than 3KD induced the epithelial cells to secrete IL-8 through the ERK pathway^23^. This further proved the basic influence of small molecular metabolites in FBS on cells^19^, which is often overlooked in practice. Therefore, IL-8 could be used as a marker for selecting serum before using the various available brands of FBS.

In this article, we just focus on the effects of serum on epithelial cells. Epithelial cells such as HCT-8 and HT-29 are known to play important roles in innate immunity^24^. Rather than exogenous pollutants like LPS, endogenous substances can stimulate epithelial cells to secrete certain inflammatory cytokines. In fact, we tested more than six kinds of epithelial and blood cancer cells. Our data showed that while some endogenous substances in FBS caused epithelial cells to secrete certain inflammatory factor, no effect on immune defense-related blood cancer cells was detected. One possible explanation is that blood cells received endogenous metabolites for a long time to form immune tolerance^25,26^.

In summary, prior to the emergence of serum substitutes with moderate prices and fixed components, establishing a relatively uniform standard of quality for FBS used in cell cultures to improve the repeatability of experimental results in scientific studies would be a low-cost, highly effective, and urgently needed means for promoting the development of scientific research.

## Supplementary Information

Supplementary Information 1.

Supplementary Information 2.

## Data Availability

The data used to support the finding of this study are available from the corresponding author upon request.
