# Supplementary material for: Fetal bovine serum, an important factor affecting the reproducibility of cell experiments
Source: Sci Rep. 2023 Feb 2;13:1942. doi: 10.1038/s41598-023-29060-7 (PMC9894865; doi:10.1038/s41598-023-29060-7)
Supplement: Supplementary file 1 — Supplementary Information 1. [file 41598_2023_29060_MOESM1_ESM.pdf]

Fetal bovine serum, an important factor affecting the reproducibility of cell experiments

Shuai Liu[1, 4], Wei Yang[2, 4]✉, Yunlei Li[3], Changqing Sun[1]✉

Supplementary dataset file legend

Differential metabolites of 1S-3S-2A-7A *vs* 4S-5A-6N-8N. Using non-targeted metabolomics to analyze the endogenous metabolites in the IL-8 stimulation group (4S, 5A, 6N, and 8N) and the IL-8 non-responsive group (1S, 2A, 3S, and 7A). Compared to the IL-8 non-responsive group, the 12 up-regulated metabolites in the IL-8 stimulation group were MEDP0338, MEDL00392, MW0103657, MW0113851, MW0055219, MW0168281, MEDP1337, MW0141191, MW0103392, MW0151467, MW0014067, and MW0112125, and the 19 down-regulated metabolites were MW0014926, MW0133678, MEDP0085, MW0107567, MW0112074, MW0147983, MW0007279, MW0108268, MW0006389, MW0005716, MW0106259, MW0150412, MW0108267, MEDP1294, MEDP0298, MW0012942, MW0107844, MW0162477 and MEDN0036.

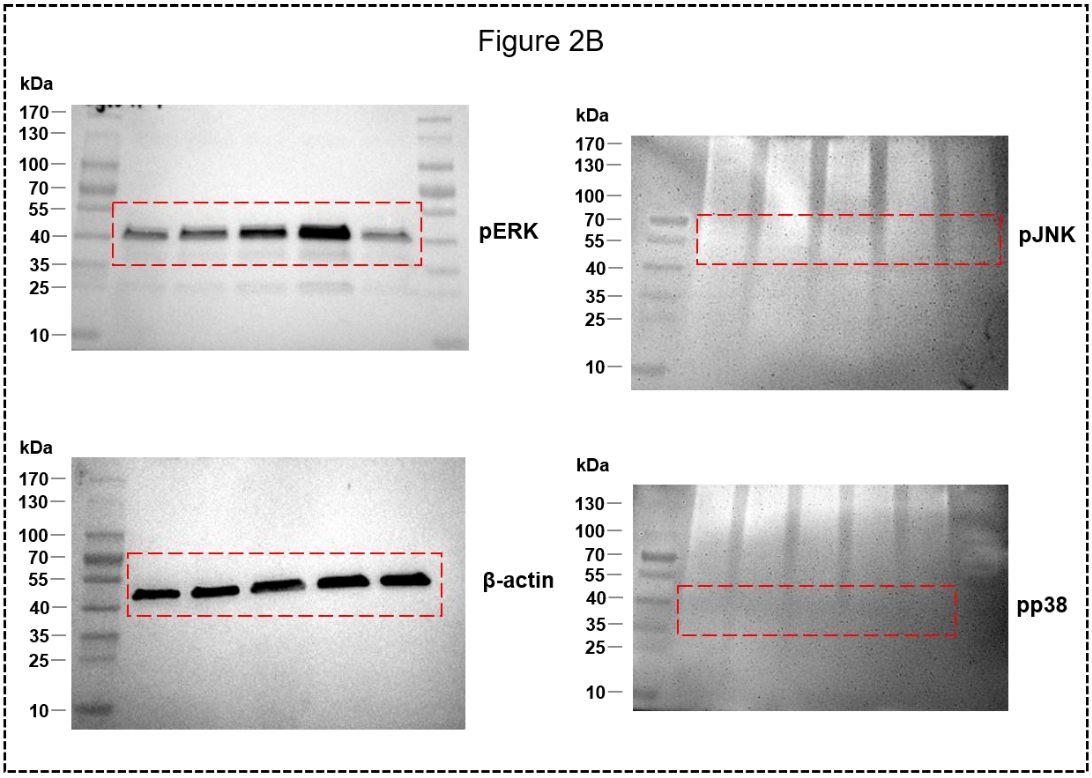

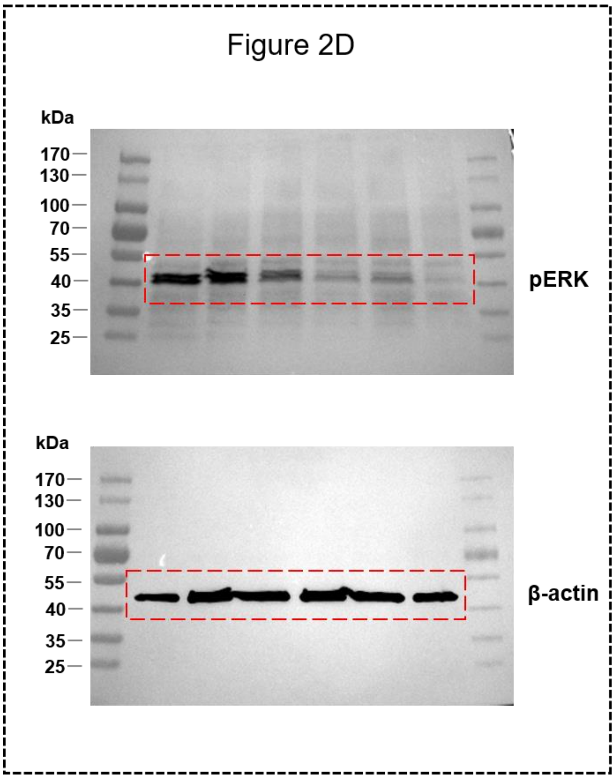

Supplementary Figure 1. Full gel images for those cropped in the paper figures. The specific bands shown in the figures are highlighted by red boxes.
